# 

**Published:** 1818-06

**Authors:** 


### INDEX.

A

ABDOMEN, on wounds of the 321
———————— 509
Abdominal diseases, cases of 254
———— tumours, account of a paper on 295
Abscesses, extensive, new mode of treating 272
Acid, nitro-muriatic, useful in the cure of diseases 51
Ætiology of tropical fever, remarks on the 81
Affections, intestinal, cases of 68
Amputation at the shoulder-joint, case of 13
Aneurism, aortic, case of 87
————, popliteal, on the operation for 201
————, aortic, complicated with dropsy 340
*Angina pectoris*, oxygen gas used in 297
Apoplexy, pulmonary, case of 527
————, cutaneous, case of 528
Apothecaries, associated, resolutions of 266
Arsenic, remarks on 34
————, cases of poisoning by 35
————, analytical experiments on 39
————, various tests for the discovery of 40

B

Balfour's, Dr. cases of gout 191
————, Dr. on the internal use of the nitrate of silver 464
Bailey, Mr. on the use of belladonna 136
Bancroft's, Dr. sequel to an essay on yellow fever 111
Belladonna, observations on the use of 136
Bell's, Mr. Charles, surgical observations 217
Bibliography 88
Biliary duct, case of enlargement of the 335
Black's, Dr. dissections of habitual drunkards 295
———— case of medullary tumour 296
Blackadder, Mr. on *phagædena gangrenosa* 331
Bladder, remarks on an operation on the 74
Blagden's, Mr. case of fatal hæmorrhage from extracting a tooth 55
Blood, analysis of the 164
Blood vessels, on injuries of the 214
Bones, on injuries of the 212
BOOKS, NEW, ANNOUNCED.—Dr. Palmer's Translation of Kreysig on Diseases of the Heart, 87. Mr. Cook's Address to British Females on the Moral Management of Pregnancy and Labour, 179. Dr. Charles Parry's Appendix to Dr. Parry's Experimental Inquiry into the Nature of the Arterial Pulse, 179. Dr. Crichton on the Vapour of Boiling Tar, 180. Dr. Gordon's Engravings of the Human Skeleton, 180. Dr. Vetch on the Non-contagious Nature of Yellow Fever, 180. Mr. Foster on Pestilential Fever, 180. Mr. Blackadder's Observations on Phagedæna Gangrenosa, 356. Mr. Ware on Diseases of the Eye, 356. Mr. Gray's Botanical Work, 356. Mr. Newnham's Essay on the Symptoms, Causes, and Treatment of *Inversio Uteri*, 444. *M. La Baume's* Observations on the Properties of the Air Pump, Vapour Bath, &c. 532
BOOKS REVIEWED.—Dr. Mayo's Remarks on Insanity, 26.—Mr. Marshall's Remarks on Arsenic, 34.—Dr. Majendie's Elementary Summary of Physiology, 44. Medico-Chirurgical Transactions, Vol. 8. Part I. 51. Dr. Bancroft's Sequel to an Essay on Yellow Fever, 111. Mr. Bailey's Observations relative to the Use of Belladonna, 136. Mr. Carlisle's Essay on the Disorders of Old Age, 138. Dr. Crichton's Account of Experiments with the Vapour of Boiling Tar, 141. Dr. Gordon's Outlines of Lectures on Human Physiology, 148. Dr. Hutchison on the Operation for Popliteal Aneurism, 201. Mr. S. Cooper's Dictionary of Practical Surgery, 206. Mr. Hennen's Observations on Military Surgery, 208, 310, 507. Mr.

Charles Bell's Surgical Observations, Part IV. 217. Dr. Robertson's Thesis *De Dysenteria Regionum Calidarum*, 228. Surgical Essays, by Mr. Cooper and Mr. Travers, 241. Mr. Moore's History and Practice of Vaccination, 283. Dr. Dewar's Account of Epidemic Small-pox at Cupar, 283. Transactions of the Association of Fellows and Licentiates of the King's and Queen's College of Physicians in Ireland, 293. Dr. Marshall Hall s Principles of Diagnosis, 303. Mr. Kirby's Observations on the Treatment of Hæmorrhoidal Excrescences, 308. The Dublin Hospital Reports and Communications, 322. Mr. Home Blackadder's Observations on *Phagedæna Gangrenosa*, 381. Mr. Kerr's Observations on the Harveian Doctrine of the Circulation, 392. Dr. Ewing's ditto, 392. Dr. Marcet on the Chemical History and Treatment of calculous Disorders, 401, 493. Dr. Lucae's *Anatomische Untersuchungen der Thymus*, &c. 406. Dr. Spurzheim on Insanity, 412, 497. Dr Uwins's Modern Maladies and Present State of Medicine, 476. Dr. Yule's Observations on the contagious Fever at Edinburgh, 480. Dr. Percival on the deleterious and medicinal Effects of Green Tea, 481. Dr. Cheyne on the Virtues of James's Powder, 483. Dr. Prout on the Effects of Remedies on the Urinary Secretion, 485. Dr. Walker's Reply to James Moore, 487. Dr. Vetch's Observations on the Treatment of Ophthalmia 514
Boyd's, Dr. history of occurrences on board the Gorgon 16
Boyle's, Mr. observations on large doses of submuriate of mercury 197
Brain, fatal case of concussion of the 109
Brookes's, Dr. case of *hydrocephalus internus* 297
——— on oil of turpentine in Melæna, 299
Browne's, Dr. history of a wound in the neck 335

C

Calculi, urinary, on the symptoms produced by 401
——— different species of 403
Calomel, on the advantages of large doses of 359
Cancer, soft, observations on 217
———, cases of 218
Carditis, practical observations on 473
Carlisle, Mr. on the disorders of old age, 139
Cheyne's, Dr. report of the Hardwicke fever hospital 322
———, case of jaundice 332
———, on the virtues of James's powders 335
——— 483
Children, diseases of, report of 281, 379
Circulation of the blood, observations on 392
Clifton dispensary, report of diseases in the 530
Colles, Dr. on the varus or club-feet 327
——— on the causes of *trismus nascentium* 333
Consumption, efficacy of vapour of tar in 144
Contagion not the cause of yellow fever, 126
Cooper's, Mr. S. case of lithotomy 54
——— dictionary of practical surgery 206
———, Mr. Astley, surgical essays 241
Correspondence, 89, 180, 268, 356, 444
Crampton's, Mr. case of rupture of the stomach 56
———, Dr. case of ulceration of the stomach 294
——— on hydrocephalic fever 301
——— case of periostitis 336
Crichton's, Dr. experiments with the vapour of boiling tar 141
Cunningham's, Mr. case of concussion of the brain 109
——— new mode of treating extensive abscesses 272
——— on scruple doses of calomel 359
Cusack's, Dr. case of removal of a tumour 330
Cynanche laryngea, case of 1

D

Death, sudden, case of, with appearances on dissection 169
Delirium subdued by opium 170
Diagnosis, on the principles of 303
Dickinson, Mr. on the nosology of fevers 357
Dickson's, Dr. remarks on the ætiology of tropical fever 81
Dictionary of practical surgery 206
Digestive organs, on the disorders of the 304
Digitalis extensively used, with success 80

### INDEX.

Diseases and casualties in London 90
——— of old age, essay on the 139
Dislocations, observations on 242
Dispensary for children, report of the 281, 379
Dropsy and apoplexy, case of 300
Dropsy by conversion of disease from the skin 334
Drunkards, habitual, dissections of 295
Dublin, on the epidemic fevers of 302
Dysentery, symptoms and treatment of 21
———, appearances on dissection in a case of 171
———, tropical, history of 231
Dyspepsia caused by hepatic affection 450

E

Ear, on injuries of the 316
Eight's, Dr. case of hydatids in the uterus 352
Elephantiasis, remarks upon 168
Enteritis, case of, with dissection 185
Epilepsy, cases of 421
Epiphises, separation of, in adult age 176
Eruption, tubercular, case of 170
Essays, surgical, by Mr. Cooper and Mr. Travers 241
Ewing, Dr. on the Harveian doctrine of the circulation 392
Excrescences, hæmorrhoidal, cases of 308
Eye, case of punctured wound of the 77
—— description of the anatomy of 150
——, case of injury to the 316

F

Face, belladonna useful in painful disorders of the 137
——, on injuries happening to the 316
Febrile diseases, cases of 257
Fever, yellow, sequel to an essay on 111
——, West India, on the treatment of 167
——, infantile remittent, observations on 370
Fevers, epidemic, of Dublin, a paper on the 302
Finger, punctured wound of the, succeeded by a nervous affection 59
Fish poison, remarks on cases of poisoning by 97
Flexure, sigmoid, of the colon, case of stricture in the 261
Forbes's, Dr. John, medical reports 93
Fouquier's, Dr. case of inflammation of the liver 278
Fracture of the skull, severe case of 168
Fungus hæmatodes, cases of 61

G

Gall and Spurzheim's, Drs. reply to the Edinburgh Reviewer 266
Gangrene, hospital, observations on 311
Gastrotomy, successful performance of 76
Glasgow, prevalence of contagious fever at 518
Goodeson, Dr. on the remittent fever at Corfu 329
Gordon's, Dr. outlines of lectures on human physiology 148
Gorgon hospital ship, occurrences on board the 16
Gout, case of, with dissection 67
——, cases of, with observations 191
Graham, Dr. on the contagious fever at Glasgow 518
Grattan's, Dr. case of chronic rheumatism 301
Gun-shot wounds of the knee joint, report of 222
Guthrie, Mr. on the treatment of the venereal disease 510

H

Hæmorrhage, fatal, from the extraction of a tooth 55
Hall's, Dr. principles of diagnosis 303
Hardwicke fever hospital, report of the 322
Head, belladonna useful in painful disorders of the 137
——, cases of injury of the 226
Heart, cases of diseases of the 491
Hemiplegia, case of 79
Hennen, Mr. on some important points in military surgery 208
Hepatic disease, influence of, on the uterus 296
——— affection the cause of other diseases 445
Hernia, observations on 331
Hospitals, on the arrangement and police of 208
——— gangrene, observations on 311
———, city, report of 355
Hutchison, Dr. on popliteal aneurism 201
Hydatids in the uterus, case of 352
Hydrocephalic fever, case of 302
*Hydrocephalus internus*, case of 297
Hydrogen an acidifying principle 460
Hydrophobia, observations on 424
Hydrothorax, case of, with remarks 427

I & J

Insanity, case of 27
———, method of cure adopted in 30
Intellect, observations on the 47
Intestinal affections, cases of 68

Uterus, functions of the, influenced by hepatic disease 296
——, case of hydatids in the 352
Uwins, Dr. on the present state of medicine 476

V

Vaccination, on the history and progress of 283
Varus, observations on 327
Venerable, H.M.S. reports of the state of health in 93
Venereal disease, on the treatment of the 510
Ventriloquism, remarks on 49
Veitch, Dr. on the non-contagious property of the yellow fever 388
Vision, observation on the philosophy of 45

W

Walker, Dr. J. in reply to Mr. Moore 347
Walker's, Dr. J. reply to James Moore 487
Wardrop's, Mr. case of nervous affection 59
Water, cold, injection of, in hæmorrhoids 529
Webster's, Mr. medical and pathological observations 181
Williams's, Mr. case of peritonæal inflammation 377
Woodville, Dr. biography of 348
Wound, punctured, succeeded by a nervous affection 59
——— of the head, case of 160

Y

Yellow fever, on the non-contagious property of 388
Yule, Dr. on the contagious fever at Edinburgh 480

PRINTED BY W. THORNE, RED LION COURT, FLEET STREET.

